# Stimulation Induced Changes in Ratio Scaling Between and Within Hemispheres

**Published:** 2022-01-07

**Authors:** Tracy Kretzmer, Mark Mennemeier

**Affiliations:** 1 James A. Haley Veterans Hospital Tampa, USA; 2 Department of Neurobiology and Developmental Sciences, The University of Arkansas for Medical Sciences, USA

**Keywords:** ratio scaling, magnitude estimation, psychophysics, t-scope study, hemispheric integration

## Abstract

**Objectives:**

This paper examines if ratio scaling, the principle behind the psychophysical Power Law, is similarly performed by the left and right cerebral hemispheres and how magnitude estimates derived in each hemisphere are integrated.

**Method:**

Three models of hemispheric integration were tested (dominance, summation, and inhibition) using a cross-modal matching procedure in right-handed, male subjects. Visual stimuli were presented to one or both hemispheres using a tachistoscopic method to test each model. Olfactory stimuli were also presented to one or both nares (hemispheres) to test the dominance and summation models.

**Results:**

A dominance model was not supported as there was little difference in ratio scaling between hemispheres for either visual or olfactory stimuli. A summation model was supported for olfactory but not visual integration. Inter-hemispheric inhibition did not account for hemispheric integration.

**Conclusions:**

The most interesting findings stemmed from a comparison of experimental conditions within rather than between hemispheres. Ratio scaling parameters, the sizes of the exponents and constants, appeared to be driven by the amount of stimulation provided to a hemisphere - a greater amount being associated with higher exponents and lower constants. Variability in ratio scaling, how well data fit power functions, corresponded to whether the hemispheres received equal amounts of stimulation - equal stimulation producing a better fit than unequal stimulation. We conclude that stimulus induced cerebral activation influences the form of power functions; whereas equivalency of stimulation between hemispheres influenced the fit.

## Introduction

Ratio scaling is a technique used in psychophysics to quantify how people experience the intensity of sensory stimulation. Magnitude estimation is one method of ratio scaling. Magnitude estimation requires subjects to rate, such as with numbers, the intensity of sensory stimuli that lie above the sensory threshold (i.e., supra-threshold stimuli). Alternative methods of ratio scaling may require subjects to reproduce stimulus magnitudes from memory (magnitude production) or to match the intensities of different types of stimulation such as loudness and brightness (cross-modal matching) [1]. The current study used cross modal matching, turning a dial to match the intensities of visual and olfactory stimulation, as the method of ratio scaling.

Ratio scaling is the principle behind Stevens’ Power Law [1]. The power law holds that “equal stimulus ratios produce equal sensation ratios”. Ratio scaling is expressed mathematically as a power function. A power function is a log-log plot. Power functions summarize the ratio at which perceptions of change in stimulus magnitudes “grow” in response to the ratio of change in the objective magnitudes of a sensory stimulus. The power law applies to many different types of magnitude estimates; not only those for sensation but also those for perceived motor movement and even social judgements like judging the severity of a crime. The ubiquitous application of Steven’s Power Law suggests that ratio scaling is an organizing principle by which the peripheral and central nervous systems interact to construct mental representations of stimulus magnitude in order to make magnitude judgements [2,3]. The current study focused on visual judgements of stimulus size and olfactory judgements of smell intensity.

Ratio scaling can be characterized for a given type of sensory stimulation, i.e., a perceptual continuum such as a range of tactile, auditory, visual or olfactory stumuli that vary in magnitude, by the form of the power function – the value of its exponent and constant [1]. Power functions are derived by log-transforming both the estimates of stimulus magnitude and the measures of physical magnitude to make them linear. The estimates are then regressed on the physical measures of magnitude to yield a regression equation. For example, numbers used to rate the loudness of different sounds are log-transformed and regressed on the log-transformed, physical measures of the sounds in decibels. The exponent of the power function is equal to the slope of the regression line and the constant of the power function is equal to the y-intercept [1]. The r^2^ value is a measure of variability; indicating how well the data fit a power function. Power function parameters, the exponent, constant and r^2^ values, are convenient summary variables that can be used to examine differences in ratio scaling between perceptual continua, individuals, groups, or experimental conditions. The current study compared power function parameters constructed by each cerebral hemisphere under different experimental conditions.

Alterations in ratio scaling have been linked to unilateral brain injury, particularly in patients with unilateral right hemisphere injury and left spatial neglect [2–8]. Spatial neglect is defined as the failure to report, respond, or act upon stimuli located contralateral to brain injury [9]. Spatial neglect is associated with damage to a fronto-cingulo-parieto-temporal network implicated in spatial attention [9–11]. While spatial neglect is most common, severe, and persistent following right hemisphere lesions; spatial neglect also occurs after left hemisphere injury [12]. Ratio scaling has been shown to be altered in patients with unilateral left hemisphere injury but not necessarily in association with spatial neglect from left hemisphere injury [13]. In fact, alterations in ratio scaling after right hemisphere injury are not exclusive to patients with left spatial neglect or even dependent on the presence of spatial neglect [3]. Rather, alterations of ratio scaling appear to be greatly exaggerated in patients with spatial neglect. Therefore, patients with spatial neglect provide a good example of how brain injury alters ratio scaling.

Patients with right hemisphere injury and neglect produce power functions with lower exponents and higher constants, than either normal control subjects or patients without neglect [3]. A decreased exponent and an increased constant suggest a restricted range of magnitude estimates, such that estimates for the smallest stimuli in the range are relatively overestimated and estimates for the larger stimuli in the range are underestimated [14,15]. Additionally, magnitude estimates in neglect patients are more variable, as indicated by lower power function r^2^ values, than those produced by normal patients or patients without neglect [3]. As mentioned earlier, ratio scaling can also be altered by left hemisphere injury where the exponent is also decreased but the constant may either be increased or decreased relative to normal subjects.

One review of neurophysiological and neuroimaging studies in both humans and animals [16], posited a common “magnitude system” for estimates of space, time and quantity localized to the inferior parietal cortex of the right hemisphere. This is the same region that when damaged is most likely to produce spatial neglect. Quantity judgments, correspond to a class of perceptual continua (prothetic) that conform to ratio scaling [17]. In contrast to the conclusion of the aforementioned review, one fMRI study of comparative magnitude estimates concerning number, size and luminance in normal subjects [18] found evidence for bilateral cerebral activation localized to the intraparietal sulci and the precentral and occipitotemporal areas. The conclusions from both manuscripts converge with those from brain injured subjects to suggest that each cerebral hemispheres engages in ratio scaling. What is unclear is how the magnitude estimates for each hemisphere are normally integrated to yield a unitary percept. The purpose of the current study, therefore, was to test three models by which magnitude estimates formed in each hemisphere might be integrated in normal, healthy subjects.

The first model is a right hemisphere dominance model for ratio scaling which predicts that power functions generated by the right hemisphere will be qualitatively different than those generated by the left hemisphere. Because exponents are decreased and constants increased following right hemisphere injury, it is predicted that the right hemisphere normally generates higher exponents and lower constants than the left hemisphere. This is plausible because the exponents and constants derived from most patients with left brain injury, who have an intact right hemisphere, are not different from normal subjects [3,8]. In other words, the “normal” exponent and constant for a given continuum may reflect a greater contribution of the right than left hemisphere.

Alternatively, the two hemispheres may perform ratio scaling equally well and integration may follow a summation model. Stevens [19,20] observed that when sounds are presented simultaneously to both ears, versus a sound presented to one ear alone, subjects report, not a doubling of the loudness, but a fractional increase in loudness, a summation, that depends on initial stimulus intensity. Summation is denoted by an increase in the size of the power function constant without a change in the size of the exponent. Ratio scaling is not altered but rather a general increase in perception of stimulus intensity occurs. A summation model of hemispheric integration would be consistent with one study of normal subjects [18] but not with data from brain injured patients which is more consistent with a dominance model for ratio scaling.

Inter-hemispheric inhibition provides a third model of how estimates of stimulus magnitude might be integrated between hemispheres and how perception of stimulus magnitude is altered following left and right hemisphere injury. The two cerebral hemispheres are viewed as mutually inhibitory of each other [21,22]; however, it is not clear which hemisphere exerts a greater inhibitory influence when the stimulus environment creates competition between hemispheres. Heilman [22] proposed that the right hemisphere is dominant for attention because it is activated by novel visual stimuli in both the left and right hemi spatial fields, whereas the left hemisphere is activated only by stimuli in the right hemi spatial field. In contrast, Kinsbourne [21] argued that the left hemisphere is normally more inhibitory of the right than the right is of the left.

Inter-hemispheric inhibition may be magnified by unilateral brain injury. For example, sensory extinction occurs following unilateral brain injury when perception of a stimulus delivered to the contralesional side of the body is obliterated by a competing stimulus delivered simultaneously to the ipsilesional side of the body [9]. Extinction is common in patients with neglect, and it almost certainly involves inhibition of one hemisphere by the other. A PET study of competing stimuli presented in both visual hemifields [23] showed reduced activation of striate and extrastriate visual cortex when competition between hemispheres was present and greater activation in the stimulated hemisphere when competition between hemispheres was absent. Further, a repetitive transcranial magnetic stimulation (rTMS) study of visual extinction found occipital rTMS led to a large number of misses for contralateral targets, presented both singly and bilaterally, whereas parietal rTMS did not cause misses on single stimuli but did lead to a large number of contralateral misses for bilateral stimuli [24]. Damage to the fronto-cingulo-temporo-parietal network in one hemisphere may have an effect similar to that of rTMS, except that, when both hemispheres are stimulated at the same time, the damaged hemisphere is also inhibited by the intact hemisphere. It follows, that extinction may not be confined to threshold stimuli but may apply as well to ratio scaling for suprathreshold stimuli. In a case study of neglect, Chatterjee and colleagues [25] found the power function relationship for estimates of weights lifted with the hand contralateral to brain injury was obliterated when the patient also lifted weights with the ipsilateral hand. In other words, ratio scaling was extinguished by activation of the intact hemisphere.

Whereas extinction is not readily observed in normal subjects; a partial form of extinction called obscuration has been observed in normal subjects [26]. They observed that the obscuration method of Jacob Loeb provided a model of interhemispheric inhibition for normal subjects. This method used double simultaneous stimulation, as in extinction techniques, except a stronger stimulus was applied to one side of the body to induce suppression of a weaker stimulus applied on the opposite side. Healthy controls were asked to rate weights raging in size from 1 to 20 grams. These target weights were presented to either the left or right forearm unilaterally. Subjects also rated the weight of stimuli when a 40-gram weight was presented simultaneously on the opposite side of the body. Obscuration stimuli led to a reduction in estimates of weight relative to when stimuli were rated without obscuration stimuli. In other words, obscuration stimuli presented to one side of the body inhibits estimates of stimulus magnitude for stimuli presented on the opposite side of the body. As a result, they concluded that extinction and obscuration were exaggerated expressions of a normal neural mechanism but, to our knowledge, ratio scaling has never been investigated using obscuration in healthy subjects.

In contrast, the obscuration method was used in this study to test an inter-hemispheric inhibition model of the integration of ratio scaling between hemispheres. For example, the right hemisphere might normally contribute more to ratio scaling, not because it generates different power functions than the left, but because it is activated by novel environmental stimuli causing inhibition of the left hemisphere. If so, presenting obscuration stimuli to the left hemisphere should counteract this effect (cause right hemisphere inhibition), whereas presenting obscuration stimuli to the right hemisphere should enhance this effect (enhancing left hemisphere inhibition). A result in the opposite direction would favor Kinsbourne’s assertion that the left hemisphere normally exerts a greater inhibitory influence on the right than vice versa [21].

In the current study, models of hemispheric integration for ratio scaling were tested by presenting stimuli either to one hemisphere (unilateral presentation) or both hemispheres simultaneously (bilateral presentation). Two sensory modalities, visual and olfactory, were used in the study. Visual stimuli were presented using tachistoscopic methods, which allows the “loading” of sensory information more directly into one hemisphere [27]. Olfactory stimuli were presented using odor sticks. The use of olfactory stimuli takes advantage of the fact that the olfactory pathways are ipsilateral [28,29], so like the t-scope, presenting stimuli to one naris “loads” the ipsilateral hemisphere with olfactory stimulation. Subjects rated stimuli presented to one hemisphere using the contralateral hand to turn a dial in order to preserve compatibility between hemisphere presentation and hand rating. Because this study examined hemispheric contributions to ratio scaling, right-handed male subjects were recruited specifically to emphasize any lateral asymmetries that might be found. Lateralized differences in stimulus processing are exhibited to greater degrees in both males and right-handers [30–32]. A right hemisphere dominance model would be supported if, during unilateral presentations, exponents generated by the right hemisphere were greater, and constants lesser, than those generated by the left hemisphere. A summation model for hemispheric integration would be supported, if during bilateral stimulus presentations, an increase in the constant but not the exponent was observed. Finally, competing predictions based on Heilman and Kinsbourne’s models of inter-hemispheric inhibition were tested using obscuration stimuli as described above.

## Materials and Methods

### Participants

In accordance with the ethical principles of the 1964 Declaration of Helenski, this study was approved by the University’s Institutional Review Board (IRB). All participants provided written informed consent prior to study inclusion. Forty-four right-handed volunteers participated in the experiment, ranging in age from 18–35 (22.4 ± 4.8). Right-handedness was defined as a score greater than 75% on the 10-item Edinburgh Handedness Inventory [33]. All participants had normal or corrected-to-normal vision in both eyes as measured by the Lighthouse Distance Visual Acuity Test, 2^nd^ edition, eye chart. Exclusion criteria were acute psychiatric symptomology, history of neurological abnormalities, anosmia, or hyposmia.

### Apparatus and stimulus materials

#### Visual magnitude estimation

Visual stimuli to be estimated consisted of eight solid black squares ranging in size from 4.5–14.0 cm. A total of 132 stimulus slides were used in three separate paradigms: 1) bilateral, 2) unilateral, and 3) obscuration.

Stimuli were presented to participants using a Lafayette 43016 shutter control tachistoscope(t-scope) with a 100 millisecond timer to control stimulus duration and a Kodak Ektragraphic IIIe Plus slide projector with a MKO zoom (100–150 mm) lens. A black dot with a diameter of 1.0 cm was placed at the center of the screen as a fixation target. To ensure consistent head orientation, a chin rest was bolted at the opposite end of a table directly in front of the screen.

**Figure 1.**
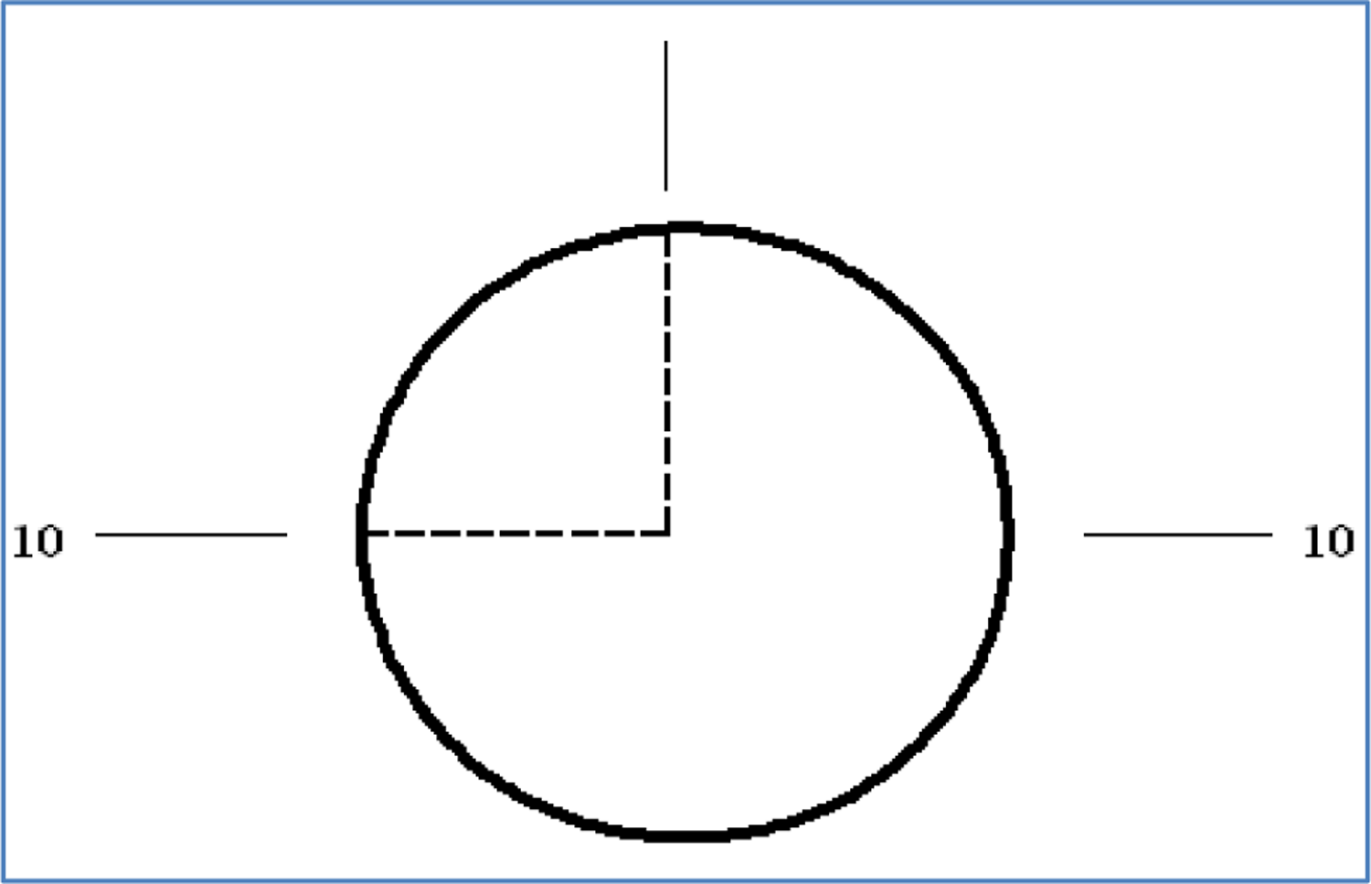
Cross modal matching

Participants were asked to rate the intensity of each visual stimulus by cross modal matching - turning a dial with their right and left hand (Figure 1). This procedure eliminates a verbal response which can activate the left hemisphere. Dial turns were recorded using a transducer that displayed a number on an LCD screen out of visual range of the participant.

#### Olfactory magnitude estimation

To prevent retronasal smelling and trigeminal excitation, the odorant phenyl-ethylalcohol (PEA) was chosen [34]. Five concentrations of PEA, ranging from 0.78%−100% were presented to each participant. Burghart’s “Sniffin’ Sticks” kit was used to dispense PEA odorants. Sniffin’ Sticks are sealed odorant containing pens used to test olfactory performance [35]. Participant responses were produced as before with a dial turn.

### Procedure

#### Visual magnitude estimation

Participants were seated upright in straight-backed chairs 113 cm in front of the projection screen with their chins in the rest. Prior to experimental trials, each participant was instructed to focus on the fixation point. They were specifically told not to deviate from fixation. To ensure fixation, the subject’s eyes were recorded from an aperture in the fixation point and monitored on a TV screen. Trials were re-administered if the participants’ gaze deviated from the central fixation point. Prior to each stimulus presentation, participants were instructed to focus on the fixation stimulus. After 500 msec the stimulus was presented for 100 msec. Participants were instructed to maintain their focus on the fixation point even during target stimulus presentation. After stimulus presentation, participants were asked to rate stimulus intensity. Redirection of centre fixation was stated between each trial. To familiarize the participants with the procedure, 16 practice trials were given prior to formal testing. Failure to discriminate either large area differences in terms of using the dial (i.e., turning the dial further for larger area trials vs. turning the dial less for smaller area trials) or verbal responses (i.e., stating whether the area box was smaller or larger than the previous slide shown) after 16 practice trials resulted in participant elimination. Two participants were excluded for this reason.

Prior to stimuli presentation, participants were instructed on cross-modality matching methodology. They were instructed to turn the dial, within a 90-degree radius, to match the size of the square with either their left or right hand. During right-handed responses, the left side of dial represented lower intensity ratings and mid-line represented higher intensity ratings. This rating scale was reversed for left-handed responses, with the right side of the dial representing lower intensity ratings and mid-line reflecting higher intensity ratings (Figure 1).

**Figure 2.**
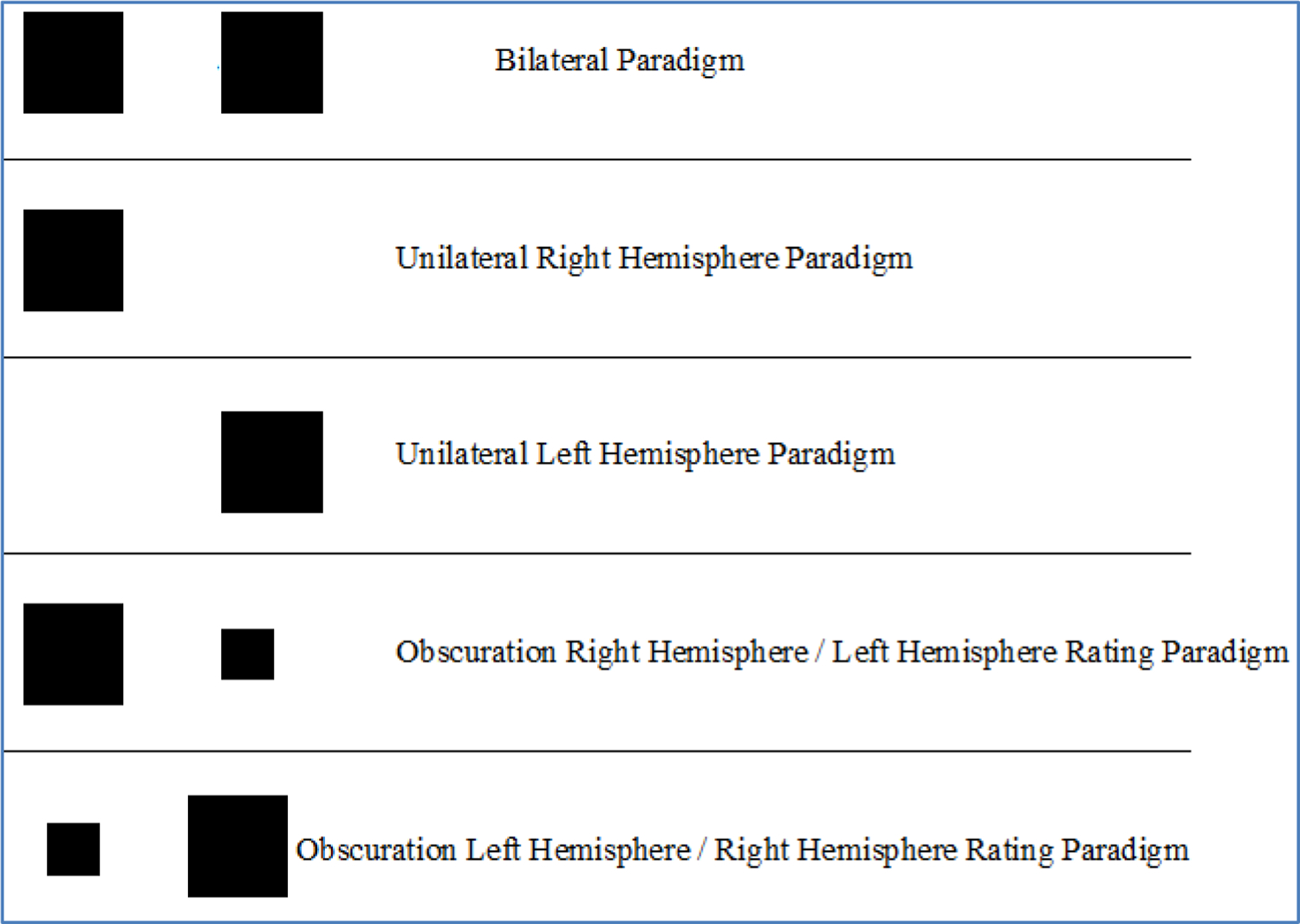
Tachistoscopic magnitude estimation paradigms

Visual stimuli were randomly presented in three separate paradigms: 1) bilateral, 2) unilateral, and 3) obscuration (Figure 2). Each paradigm was presented twice, once to record right hand responses (RHR) and once to record left hand responses (LHR). To control for order effects, RHR and LHR were counterbalanced across all stimuli, paradigms and participants. Right unilateral, left unilateral and bilateral stimuli consisted of eight area magnitudes presented randomly in three separate blocks, equalling 24 magnitude estimations each. Stimuli were projected at a distance of 4.0 cm from the center of the fixation point to the center edge of the stimuli, thus subtending a visual angle of 5°.

Obscuration trials consisted of one of six target stimuli, ranging from 4.5–8.5 cm, on one side of the fixation point and one modulus stimulus, measuring 14 cm, on the opposite side of the fixation point. Twelve slides were used, with six target-right slides and six target-left slides presented three times each (Figure 2).

#### Olfactory magnitude estimation

Participants were required to not eat or drink anything, except water 15 minutes prior to odorant exposure. Participants were instructed to rate the magnitude of each presented stimuli via cross modality matching procedures discussed above. Each of the five concentrations were randomly presented in three separate blocks with a brief resting interval of two minutes between each block. Olfactory presentations included separate bilateral and unilateral paradigms. For bilateral odor presentation each of the concentrations were placed approximately 2 cm under each nares for three seconds. To prevent environmental odor contamination, the examiner wore disposable odorless gloves. At least 30 second intervals between each trial were provided to prevent olfactory desensitization [36]. Participants were free to sample the odors as often as necessary. Responses were recorded with separate RHR and LHR procedures. For unilateral trials, odorant pens were presented to either the left or the right naris exclusively. To ensure unilateral presentation, the participant’s contralateral naris was occluded with 3M Microfoam surgical tape. Due to ipsilateral pathways, participants were instructed to respond with their contralateral hand only.

### Statistical analysis

Each participant’s log-transformed, ratings were regressed onto log-transformed, measures of actual stimulus intensity to yield separate exponent, constant and r^2^ values. A significant r^2^ value indicated that the data fit power functions. Power functions were calculated using SPSS v.11 package. Repeated measure analyses were conducted with specific contrasts to examine each model proposed using SAS v.9 package.

## Results

### Participants

Power functions not reaching significance (r^2^<0.05) were not analyzed. This excluded two participants from the visual data set and nine participants from the olfactory data set. To ensure that power functions generated from the visual and olfactory experiments accounted for roughly equal portions of variance, power functions yielding extreme r^2^ values, as defined by values greater than 1.5 times the inter-quartile range within each paradigm, were eliminated from further analyses. This resulted in a loss of three additional participants from the visual data set and one from the olfactory data set. Finally, four more participants were excluded from the visual data set due to missing values (a requirement for the repeated measures procedure). In the end, the visual data set was comprised of 29 participants (26 participants for the obscuration analyses) and the olfactory data set was comprised of 31 participants.

### Right hemisphere dominance model

The size of power function exponents and constants were not different between hemispheres during unilateral stimulus presentations for either visual or olfactory stimuli.

### Summation model

For visual stimuli paired contrasts demonstrated significant differences between right hemisphere bilateral (1.05 ± 0.34) and unilateral exponents (0.94 ± 0.29) (F=6.96, *p*=0.02) and bilateral (6.24 ± 3.88) and unilateral constants (7.69 ± 4.82) (F=4.99, *p*=0.04).

Analyses of left hemisphere bilateral trials versus left hemisphere unilateral trials demonstrated no significant differences in exponent sizes. However, significant differences, were found for the constants between left hemisphere bilateral (6.12 ± 3.44) and left hemisphere unilateral conditions (9.04 ± 6.14) (F=5.79, *p*=0.03), in the same direction as that observed for the right hemisphere.

No significant differences were found between olfactory bilateral and unilateral exponent sizes, regardless of cerebral hemisphere. Right hemisphere comparisons revealed significantly higher bilateral constant sizes (25.46 ± 11.48) than unilateral constant sizes (21.65 ± 8.74) (F=7.04, *p*=0.01). Left hemisphere comparisons also demonstrated significant differences, in the same direction, between bilateral (24.31 ± 9.64) and unilateral constant sizes (19.49 ± 8.84) (F=15.51, *p*=<0.001).

### Inhibition model

Comparative analyses did not demonstrate significant differences in either exponents or constants between right and left obscuration trials.

Further, exponents and constants for obscuration trials were compared to those for bilateral and unilateral stimulus presentation trials. Paired contrasts did not reveal differences between right hemisphere bilateral trials and right hemisphere obscuration trials (left hemisphere receives obscuration stimulus-right hemisphere receives stimulus to be rated), in either the size of the exponent or constant. However, right hemisphere unilateral trials yielded significantly lower exponents (0.94 ± 0.29) and significantly higher constants (7.69 ± 4.82) than right hemisphere obscuration trials for the exponents (1.15 ± 0.47) (F=8.71, *p*=0.008) and constants (5.90 ± 5.60) (F=4.24, *p*=0.05).

Similar contrasts for the left hemisphere revealed lower exponents (0.97 ± 0.32) and higher constants (6.12 ± 3.44) during bilateral than obscuration trials (exponents=1.07 ± 0.45, F=183.6, *p*=<0.001; and constants=6.91 ± 5.37, F=65.24, *p*≤0.001). Additionally, unilateral exponents (0.91 ± 0.35) were lower and constants higher (9.04 ± 6.14) than during obscuration trials (F=7.44, *p*=0.01) and (F=6.26, *p*=0.02), respectively.

### r^2^ correlations

Correlations between the power function r^2^ and exponent and constant values were calculated for both visual and olfactory paradigms (Table 1). For visual stimuli, in bilateral trials, r^2^ values did not share significant variance with exponent and constant values for area judgments. In contrast, both unilateral and obscuration comparisons yielded higher r^2^ values, indicating significant correlations with both exponent and constant values.

In olfactory comparisons, r^2^ values were significantly correlated with bilateral and unilateral exponents. However, with the exception of olfactory bilateral right-hemisphere, r^2^ values did not share significant variance with constant values in olfactory comparisons.

## Discussion

Three models of hemispheric integration for ratio scaling were tested in this study to further investigate how magnitude estimates are formed and integrated by the two cerebral hemispheres. A dominance model predicts increased power function exponents and decreased constants for the right hemisphere when compared to those for the left hemisphere during unilateral stimulus presentation. A summation model predicts increased constants for both hemispheres, in bilateral versus unilateral stimulus presentations. Finally, an interhemispheric inhibition model predicts altered ratio scaling in one hemisphere when the other hemisphere receives and obscuration stimulus.

Studies of ratio scaling in brain damaged patients [3,37] and in normal subjects and animal studies [16] suggest a right hemisphere dominance model. In contrast, an fMRI study [18] of normal subjects suggested bilateral cerebral activation during ratio scaling. Results from the current study; however, did not support the right hemisphere dominance model for ratio scaling. In fact, none of the findings in this study indicated a difference in ratio scaling between hemispheres. Results were surprisingly uniform even for comparisons of different stimulus combinations within a hemisphere. Therefore, the two hemispheres appear equally adept at ratio scaling.

How then does one explain the rather strong empirical support for a right hemisphere dominance model for ratio scaling? While the right hemisphere may not contribute more to ratio scaling than the left, it may be more susceptible to factors that influence ratio scaling like reduced cerebral activation and unequal stimulus presentation. The right hemisphere plays an important role in arousal [22,38] and it may be activated by visual stimuli in both the left and right visual hemifields [22,39]. A study by Coslett and colleagues [40] demonstrated that individuals with right hemisphere damage were significantly more impaired in their capacity for cerebral activation than individuals with left hemisphere damage. Their findings indicate that right hemisphere damage may result in performance deficits like neglect because the capacity for cognitive activation is limited. So, to when neglect patients demonstrate impaired ratio scaling, lower exponents and higher constants, these findings may similarly reflect a diminished capacity for cerebral activation induced by right hemisphere injury. Further work is needed to validate these assertions, but they could explain why right hemisphere damage compromises ratio scaling more than left hemisphere damage – not because the right hemisphere is normally dominant for ratio scaling but because a decreased capacity for cerebral activation compromises ratio scaling.

**Figure 3.**
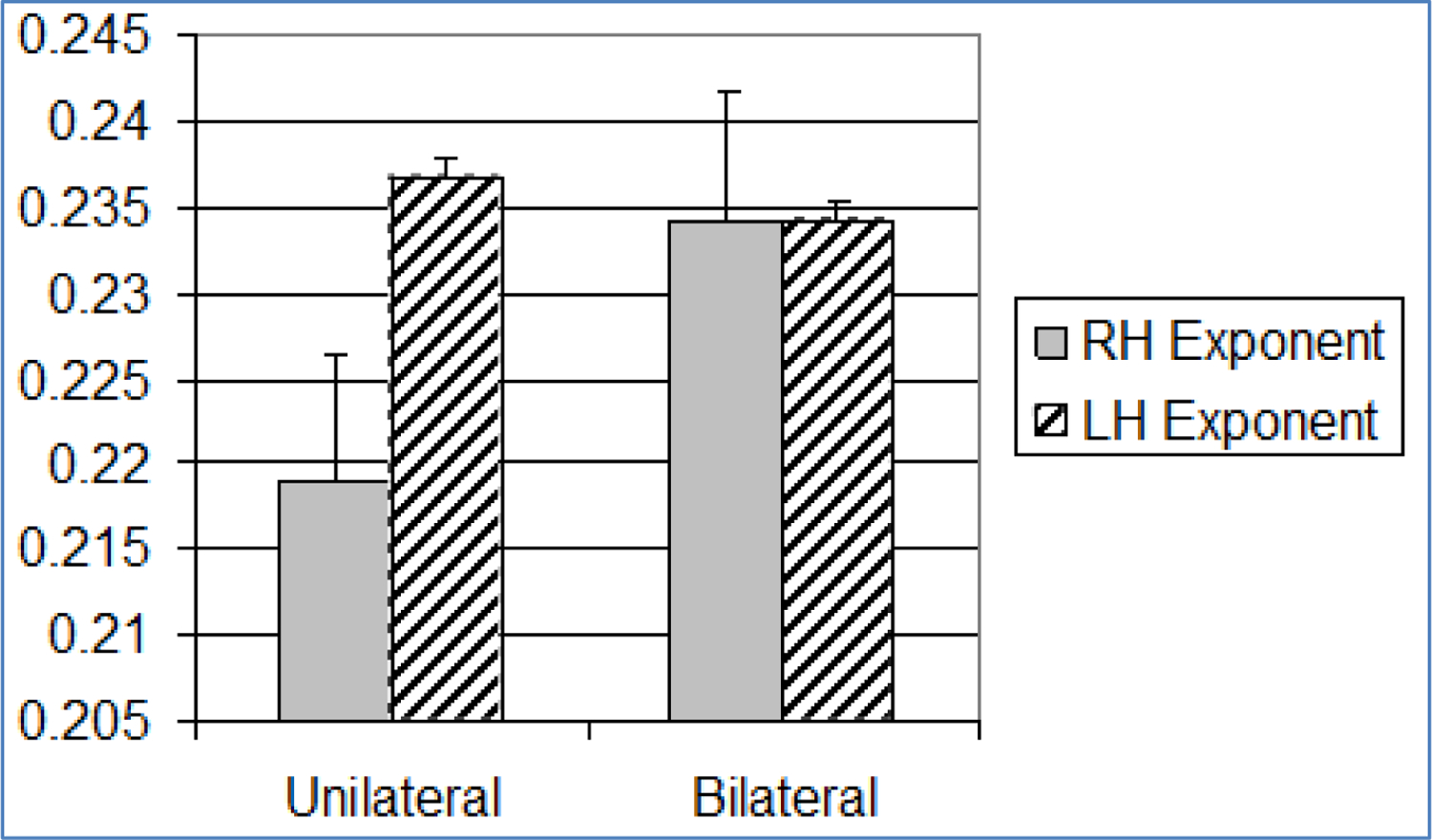
Bar graph showing mean and standard error (+1SE) of olfactory exponents across paradigm (n=31). Standard error is indicated by lines at the end of each bar. RH = Right Hemisphere, LH = Left Hemisphere

**Figure 4.**
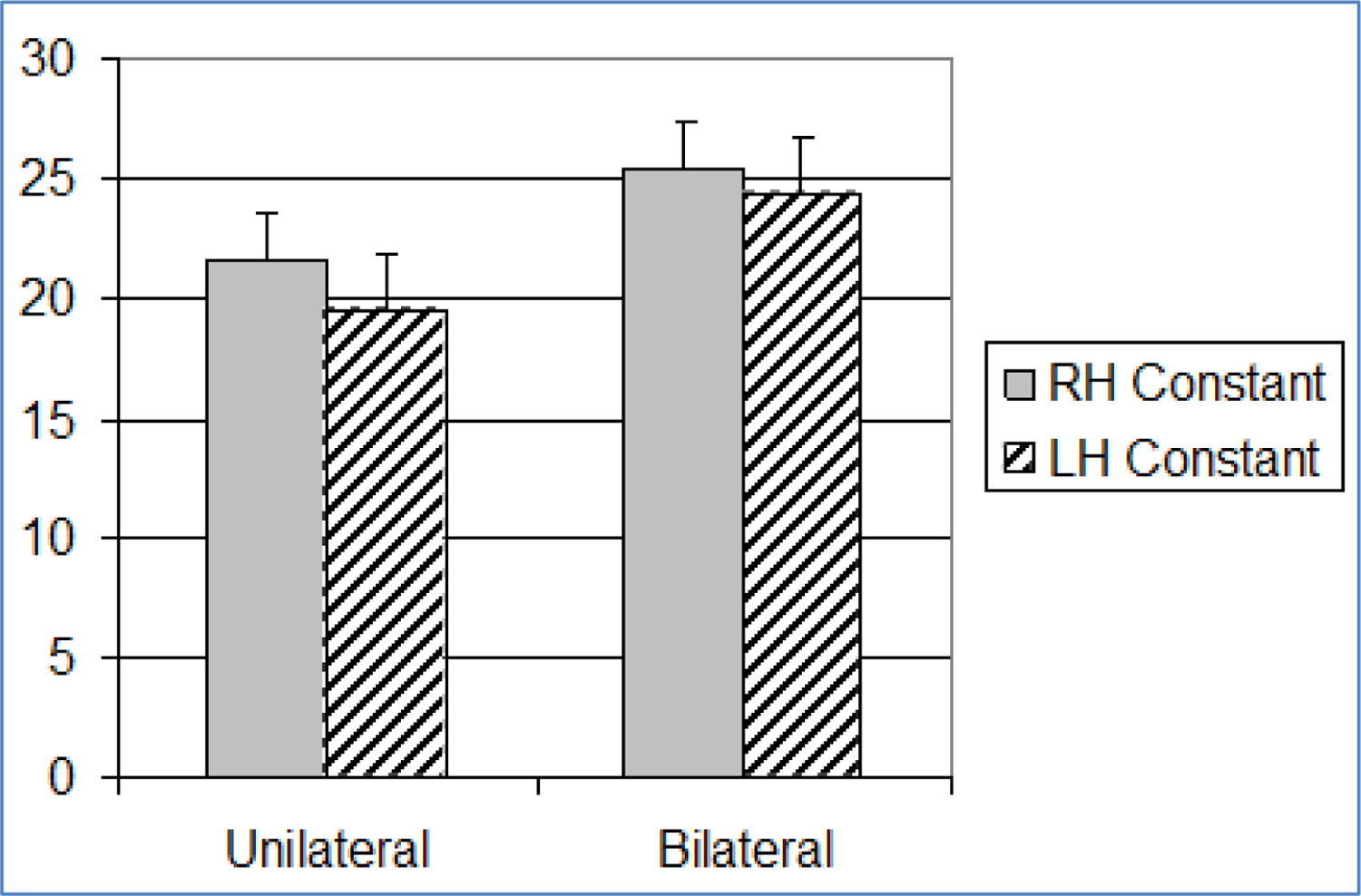
Bar graph showing mean and standard error (+1SE) of olfactory constants across paradigm (n=31). Standard error is indicated by lines at the end of each bar. RH: Right Hemisphere, LH: Left Hemisphere

The summation model tested comparisons between bilateral and unilateral stimulus presentations. It was supported only within the olfactory paradigm. Increased bilateral constants (right and left hemisphere) relative to unilateral constant sizes, without a change in the size of the exponent, reflects a general increase in perceived stimulus intensity, rather than a change in ratio scaling (Figures 3 and 4). Our results converge with early electrophysiological studies of olfaction in this regard. Early olfactory studies demonstrated that when odor was presented birhinally, a summation of impulses occurred, rather than a doubling of monorhinal impulses (Eisberg, 1935 & 1936) [41]; however, methodological differences (cross-modal matching vs. electrophysiological impulses) between the earlier studies and our approach allows us to draw only tentative conclusions. Our findings are consistent with a summation process of olfactory integration.

**Figure 5.**
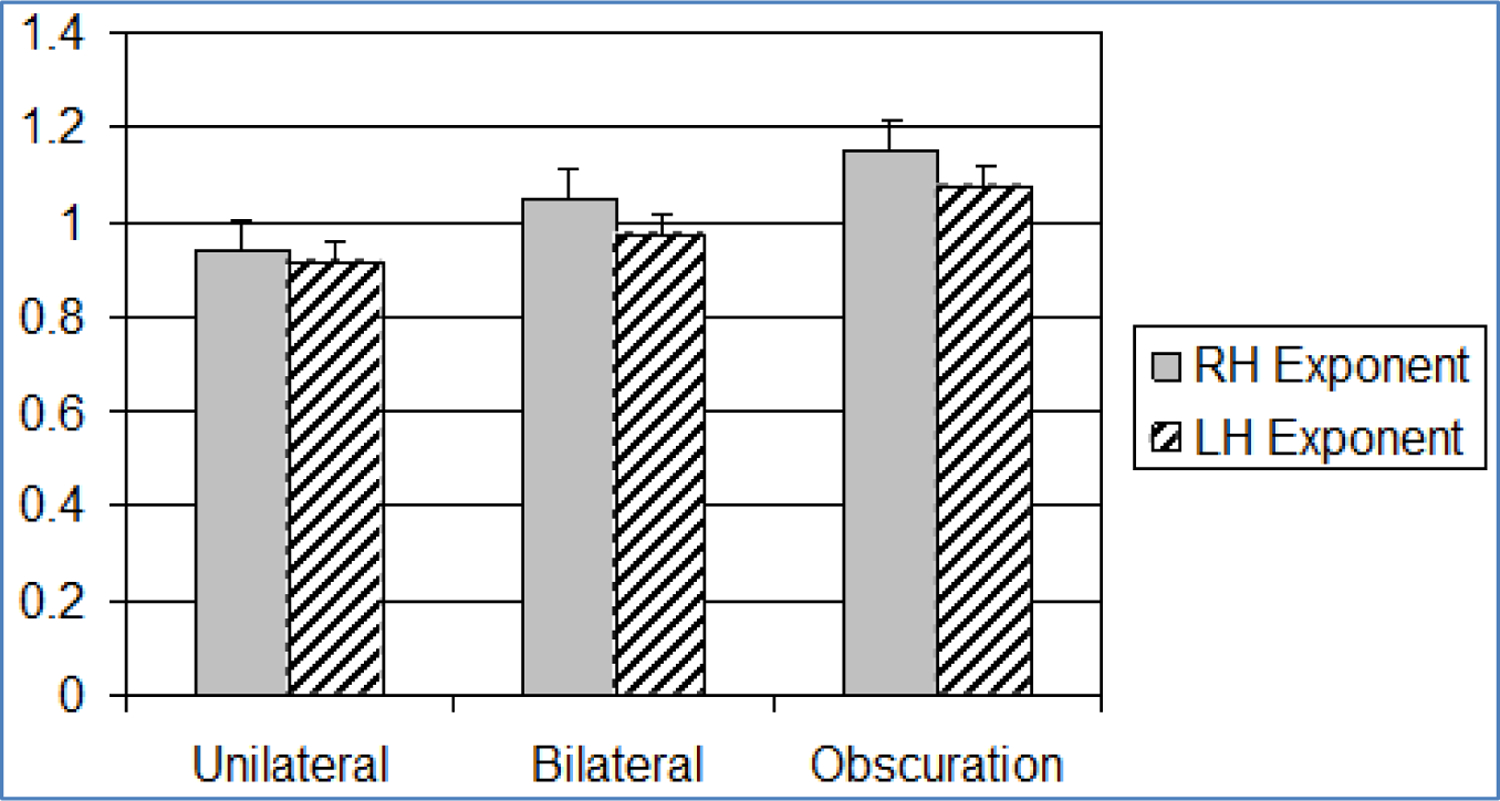
Bar graph showing mean and standard error (+1SE) of visual exponents across paradigm (n=20). Standard error is indicated by lines at the end of each bar. RH: Right Hemisphere, LH: Left Hemisphere

**Figure 6.**
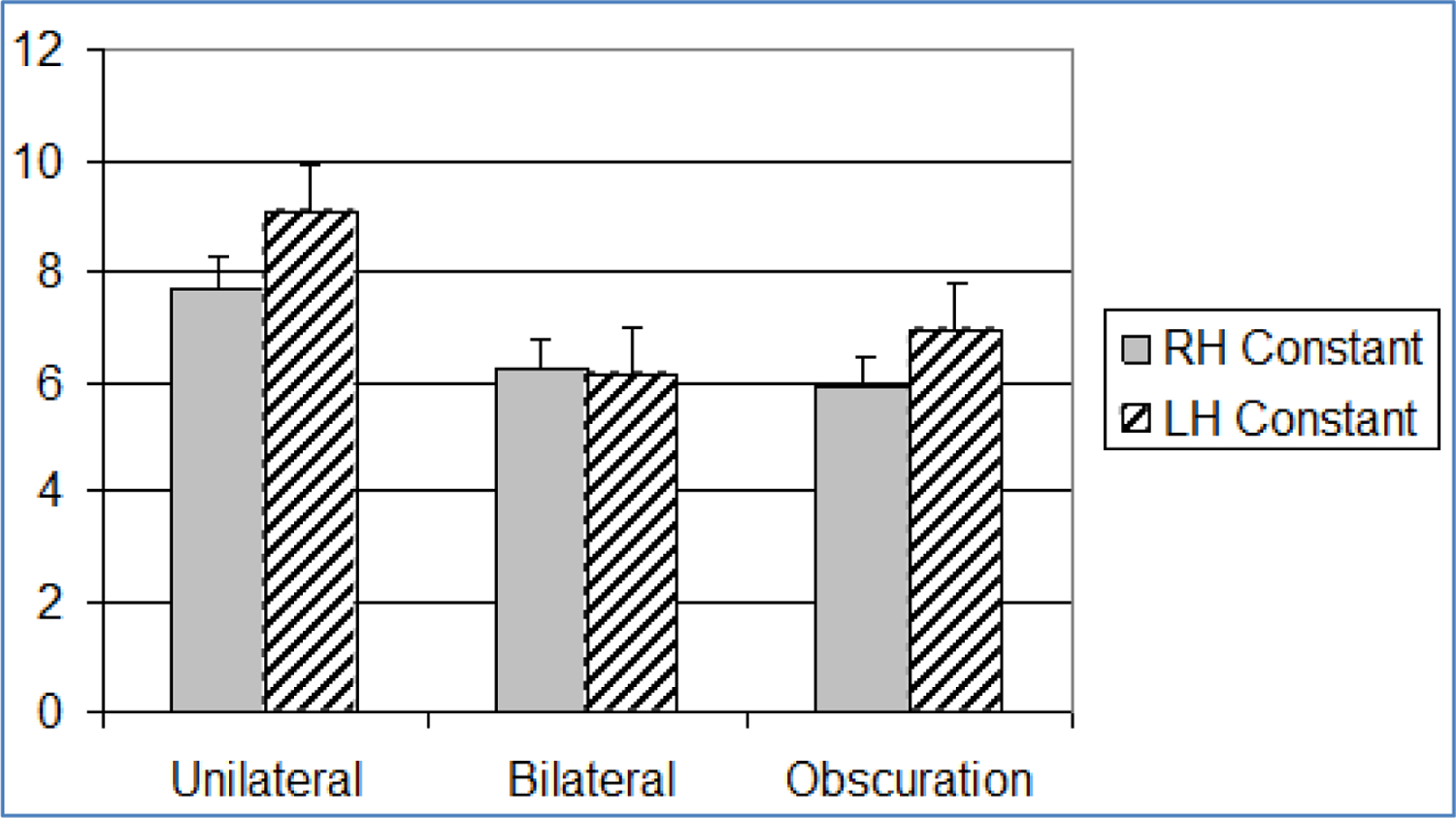
Bar graph showing mean and standard error (+1SE) of visual exponents across paradigm (n=20). Standard error is indicated by lines at the end of each bar. RH: Right Hemisphere, LH: Left Hemisphere

Integration of visual stimuli did not support a summation model. In fact, most of the significant differences in ratio scaling for visual stimuli occurred within, rather than between hemispheres. In general, bilateral visual stimulus presentations (both equal and obscuration) lead to an increase in the size of the exponent and a decrease in the size of the constant over unilateral presentations. Obscuration trials further increased the size of the exponent and decreased the size of the constant, in both hemispheres, over bilateral stimulation (Figures 5 and 6). While a few comparisons did not reach statistical significance, this trend was remarkably consistent across hemispheres and experimental conditions.

Why should bilateral stimulation drive up the size of the exponent and drive down the size of the constant? One explanation may relate to Heilman’s assertion that visual stimuli serve to activate the cerebral hemispheres. Cerebral activation may alter the form of the power function (the size of the exponent and constant) by accelerating the ratio at which perceived stimulus magnitude grows in response to changes in physical magnitude. As objective magnitude stayed constant across experimental conditions, only perceived intensity was free to change. Obscuration trials may produce even greater changes in the size of the exponent and constant because they are larger stimuli and presumably more capable of inducing cerebral activation. This conclusion is tentative because it was not predicted prior to our study but it raises an interesting and novel possibility that cerebral activation alters ratio scaling by accelerating the growth rate for perceived stimulus intensity.

**Figure 7.**
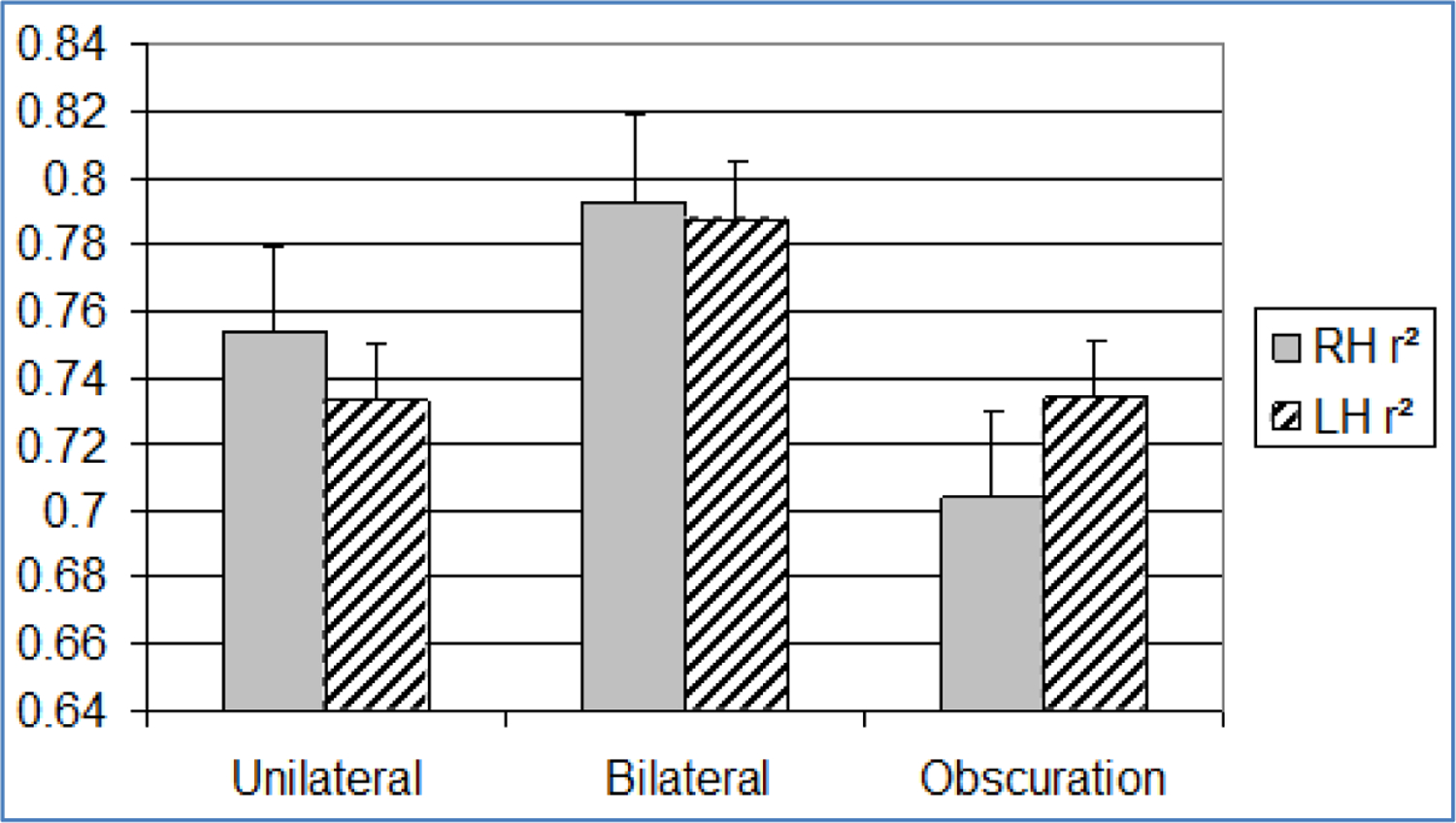
Bar graph showing mean and standard error (+1SE) of visual r^2^ across paradigm (n=20). Standard error is indicated by lines at the end of each bar. RH: Right Hemisphere, LH: Left Hemisphere

A second general finding related to variability in ratio scaling. Variability is indexed by the size of the r^2^. Whereas the size of the exponent and constant were not correlated with r^2^ during equal bilateral visual stimulus presentations, they were significantly correlated with r^2^ during unequal stimulus presentations (both unilateral and obscuration) (Table 2). We refer to these trials as unequal because the magnitude of stimulation delivered to each hemisphere is different in these conditions. Hemispheric competition in the form of unequal stimulation was associated with increased variability (low r^2^) in visual ratio scaling (Figure 7). This finding is similar to that of a neglect case study mentioned earlier [25], in which ratings by the hand contralateral to the damaged hemisphere no longer corresponded to a power function whenever the hand contralateral to the non-damaged hemisphere performed the task simultaneously.

It is important to emphasize that the size of the exponent and constant (the form of the power function) maintains a degree of independence from the size of the r^2^ (the fit of the power function). Consider for example that variability was increased during obscuration trials, but the size of the exponent was highest, and the size of the constant was lowest in this condition. Variability is also increased in the unilateral stimulation conditions, but the exponent is lowest and the constant highest. We interpret this to mean that cerebral activation alters the form of the power function and unequal cerebral activation alters the fit of data to the function.

The same influences appear to be present in studies of ratio scaling after unilateral right brain injury. Patients with left neglect have increased variability, lower exponents and higher constants than normal subjects and patients without left neglect [3,42]. Decreased cerebral activation in the right hemisphere may lead to a decreased power function exponent and increased constant, while unequal activation between hemispheres leads to increased variability in performance. Again, these conclusions are tentative, but very intriguing, because they suggest that altered ratio scaling in neglect has multiple sources of variance.

**Figure 8.**
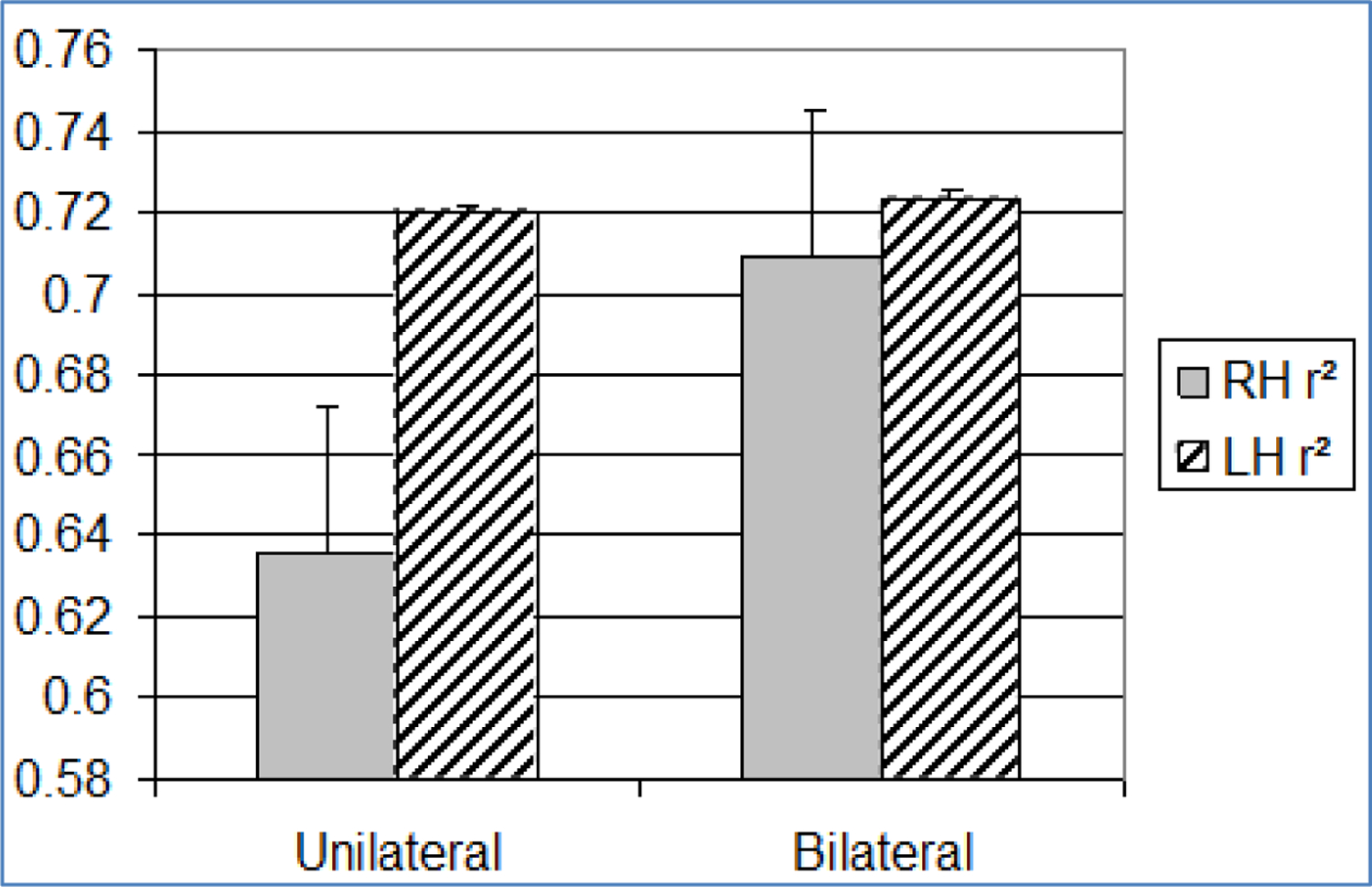
Bar graph showing mean and standard error (+1SE) of olfactory r^2^ across paradigm (n = 20). Standard error is indicated by lines at the end of each bar. RH: Right Hemisphere, LH: Left Hemisphere

Because we did not examine obscuration trials using olfactory stimuli, we do not know if increased stimulation would drive up the size of the exponent and drive down the constant as was the case for visual stimuli. However, comparisons between bilateral and unilateral olfactory stimulation did not support such an outcome. Unlike visual stimuli, bilateral olfactory stimulation led to a fractional increase in perceived intensity, rather than a change in ratio scaling. Another difference between visual and olfactory stimuli was that r^2^ correlated with the size of the exponent (but not the constant) in both unilateral and bilateral conditions (Figure 8). The findings appear to suggest that olfactory stimulation is integrated (summed) between hemispheres, whereas visual stimulation is not.

In conclusion, similarities in the way the two hemispheres construct mental representations of stimulus intensity appear to be greater than differences. Models concerning the integration of ratio scaling between hemispheres, dominance, summation and interhemispheric inhibition, may be modality specific. No model accounted for the integration of visual stimuli between hemispheres, whereas a summation model accounted for olfactory stimuli. The novel and intriguing findings of this study suggest that the form of power functions for visual stimuli are influenced by cerebral activation, whereas the fit of power functions are influenced by the equivalency of stimulation (and presumably activation) in each hemisphere.

## Figures and Tables

**Table 1.** r^2^ correlations with exponent and constant values

	Exponent		Constant	
	r^2^	*p*-value	r^2^	*p*-value
**Area Bilateral Right Hemisphere**	0.282	0.228	−0.25	0.287
**Area Bilateral Left Hemisphere**	0.263	0.262	−0.125	0.598
**Area Unilateral Right Hemisphere**	0.597	0.005	−0.495	0.026
**Area Unilateral Left Hemisphere**	0.636	0.003	−0.635	0.003
**Obscuration Right Hemisphere**	0.705	<0.001	−0.838	<0.0001
**Obscuration Left Hemisphere**	0.516	0.02	−0.581	0.007
**Olfactory Bilateral Right Hemisphere**	0.506	0.004	−0.419	0.019
**Olfactory Bilateral Left Hemisphere**	0.375	0.038	−0.18	0.333
**Olfactory Unilateral Right Hemisphere**	0.612	0.0003	−0.235	0.203
**Olfactory Unilateral Left Hemisphere**	0.631	0.0001	−0.136	0.465

**Note:** Results based on N = 20 (visual correlations) and N=31 (olfactory correlations).

**Table 2.** Mean exponents, constants, and r^2^ for group visual and olfactory paradigms

	Exponent X (SD)	Constant X (SD)	r^2^ X (SD)
**Area Bilateral Right Hemisphere**	1.05 (0.34)	6.24 (3.88)	0.79 (0.07)
**Area Bilateral Left Hemisphere**	0.97 (0.32)	6.12 (3.44)	0.79 (0.08)
**Area Unilateral Right Hemisphere**	0.94 (0.29)	7.69 (4.82)	0.75 (0.12)
**Area Unilateral Left Hemisphere**	0.91 (0.35)	9.04 (6.14)	0.73 (0.10)
**Obscuration Right Hemisphere**	1.15 (0.47)	5.90 (5.60)	0.70 (0.18)
**Obscuration Left Hemisphere**	1.07 (0.45)	6.91 (5.37)	0.73 (0.15)
**Olfactory Bilateral Right Hemisphere**	0.23 (0.10)	25.46 (11.48)	0.71 (0.16)
**Olfactory Bilateral Left Hemisphere**	0.23 (0.10)	24.31 (9.64)	0.72 (0.12)
**Olfactory Unilateral Right Hemisphere**	0.22 (0.10)	21.65 (8.74)	0.64 (0.17)
**Olfactory Unilateral Left Hemisphere**	0.24 (0.10)	19.49 (8.84)	0.72 (0.12)

**Note:** Results based on N=20 (visual correlations) and N=31 (olfactory correlations)
